# NDIEM: A Networked Drone Information Exchange Model for Heterogeneous UAV Interoperability and Communication

**DOI:** 10.3390/s26165155

**Published:** 2026-08-14

**Authors:** Bushra Younas, Jessika Delgado Ruiz, Joong-Lyul Lee, Jamshed Iqbal, Sungsoo Ahn

**Affiliations:** 1 Department of AI Convergence Engineering, Gyeongsang National University, Jinju 52828, Republic of Korea; 2024214053@gnu.ac.kr (B.Y.); joonglyul.lee@gnu.ac.kr (J.-L.L.); 2 Department of Aerospace and Software Engineering, Gyeongsang National University, Jinju 52828, Republic of Korea; 2024214517@gnu.ac.kr; 3 College of Engineering and Energy, Abdullah Al Salem University, Kuwait; jamshed.iqbal@aasu.edu.kw

**Keywords:** unmanned aerial vehicles, interoperability, data exchange model, XML schema, heterogeneous communication, MAVLink

## Abstract

**Highlights:**

**What are the main findings?**

- We propose a Networked Drone Information Exchange Model (NDIEM), an XML/XSD-based model that identifies five key data elements for information exchange among heterogeneous drones.
- We demonstrate the feasibility of NDIEM through conceptual and prototype-based validation. Experimental validation with ArduPilot, Crazyflie, and Tello EDU demonstrates schema-compliant message exchange, low latency, and practical cross-platform interoperability.

**What are the implications of the main findings?**

- The proposed NDIEM helps design a reusable foundation and develop interoperable multi-drone systems across research, educational, and operational environments.
- The heterogeneous drones can successfully exchange mission, telemetry, command, and sensor information through a structured XML/XSD representation.

**Abstract:**

The rapid adoption of small drones for applications such as surveillance, disaster response, and infrastructure inspection has increased demand for coordinated multi-drone operations, in which information exchange is essential. However, effective collaboration across different drones remains challenging due to differences in information exchange and communication protocols. This paper proposes a Networked Drone Information Exchange Model (NDIEM), an XML-based model that enables interoperability of the structural information. NDIEM defines five essential element categories: Identification, Telemetry, Command and Control, Sensor, and Mission. NDIEM can be used with protocol-specific adapters for exchanging information in multi-drone operations. The proposed model has been implemented and evaluated on three different drones (an ArduPilot-based Hexacopter, a Crazyflie 2.1, and a Tello EDU) using real sensor telemetry captured under controlled conditions, in which each platform’s onboard sensors were manipulated to generate representative telemetry variation. Experimental results demonstrate information interoperability with 0.013–0.019 ms processing overhead, 0.049–0.073 ms transformation latency per message, and XSD schema validation compliance, achieving 69.7% transformation completeness for telemetry data. These findings show that NDIEM can provide a practical and scalable foundation for software development for drone collaboration and interoperability.

## 1. Introduction

Unmanned Aerial Vehicle (UAV) systems have been adopted for critical applications such as disaster response, search and rescue, infrastructure inspection, and environmental monitoring. In practice, a single UAV platform is often insufficient to manage complex missions efficiently. The use of multiple UAVs can increase mission effectiveness by enabling increased area coverage, performing tasks in parallel, and providing operational redundancy. For example, wildfire monitoring may require simultaneous aerial mapping, environmental sensing, and real-time situational awareness, all enabled by UAVs with different capabilities and sensor configurations [1].

However, the interoperability of different UAV systems remains a significant challenge. Interoperability refers to the ability of different UAV systems to understand one another consistently across all systems and environments. As various UAVs use different hardware, software, information exchange models, and communication methods, interoperability enables them to communicate and share data reliably and in a coordinated way. Different UAV systems rely on different communication protocols and data representations for data exchange. For example, typical autopilot systems, such as ArduPilot and PX4, use the Micro Air Vehicle Link (MAVLink) protocol. In contrast, micro-UAVs, such as Crazyflie, rely on the Crazy Real-Time Protocol (CRTP). Consumer drones such as the Tello EDU use a proprietary protocol over WiFi. Although these UAVs have similar information (telemetry, position, orientation, sensor data, etc.), they use different structural and syntactic data representations, which limit efficient communication and integrated operations [2,3]. An information exchange model for drones was presented based on the National Information Exchange Model (NIEM). Still, it was an initial conceptual model that lacked experimental results and concrete use cases [4].

This heterogeneity of UAV systems leads to several operational limitations. First, every system needs its own data processing and parsing mechanism, which complicates data aggregation and interoperability. Second, operators need to use different ground control systems for each UAV in a coordinated mission, reducing operational efficiency. Third, differences in message formats, coordination systems, and mission-specific semantics make unified information exchange challenging, especially in time-intensive tasks such as emergency disaster response, rescue, and search operations [5]. Existing technologies partially address these interoperability challenges. For example, robotic operating systems (ROS 2) and data distribution service (DDS) support distributed communication for data exchange among robotic systems [6]. However, these approaches primarily focus on middleware communication and do not provide a standardized semantic structure for exchanging heterogeneous UAV information. As a result, interoperability often depends on translating the custom protocol between individual platforms, increasing the complexity of integration as the number of UAV systems increases [7].

To overcome these limitations, we propose the Networked Drone Information Exchange Model (NDIEM), a conceptual eXtensible Markup Language (XML)-based model for heterogeneous UAV environments. The proposed model separates data representation from platform-specific communication protocols and enables standardized information exchange through a unified semantic structure. In addition to native UAV protocols, NDIEM provides syntactic and partial semantic interoperability between heterogeneous UAV systems [8]. The proposed model consists of five core categories: identification, telemetry, command and control, sensors, and mission data. XML is used for its hierarchical structure, while XML Schema Definition (XSD) ensures message validation and consistency [9].

NDIEM enables platform-specific messages to be transformed into the NDIEM message format. To evaluate feasibility, a conceptual interoperability scenario involving the ArduPilot, Crazyflie, and Tello EDU platforms was developed, and experiments were performed. Results indicate that NDIEM supports standardized information exchange with low processing overhead and consistent schema-compliant communication across heterogeneous UAV environments [10,11]. The main contributions of this work are as follows: (i) the proposal of an NDIEM conceptual information exchange model for heterogeneous UAV systems; (ii) the design of a standardized XML-based information structure for UAV telemetry, sensor, command, and control data exchange; and (iii) a conceptual interoperability evaluation, demonstrating the feasibility of standardized information exchange among heterogeneous UAV platforms.

The remainder of this paper is organized as follows. Section 2 reviews related work on UAV communication and interoperability approaches. Section 3 presents the research flow and development process of the proposed model. Section 4 presents the NDIEM information model using Unified Modeling Language (UML) and XML design modeling techniques, identifying five essential data elements as part of the information model. Section 5 presents NDIEM in action. Section 6 provides the discussion. Finally, Section 7 concludes the paper and outlines future research directions.

## 2. Related Work

We review existing approaches to UAV communication and interoperability, focusing on communication protocols, middleware frameworks, and information exchange models.

### 2.1. UAV Communication Protocols

UAV systems use various communication protocols, such as MAVLink, CRTP, and DroneCAN, for their missions. UAV systems also use proprietary Software Development Kits (SDKs) based on WiFi (e.g., Tello EDU) for communication. MAVLink is a popular protocol on platforms such as ArduPilot and PX4 [12,13]. MAVLink has reliable, effective standard binary messages that encode predefined structures. However, it is based on language-specific communication descriptions, which reduces interoperability between different UAVs. CRTP is designed for small UAVs, such as Crazyflie, and enables information exchange over low-bandwidth links. However, it does not include the semantics of global positioning and standardized data representation [2]. Proprietary SDKs across different UAV systems provide flexibility but lack formal structure, extensibility, or validation. For example, Tello EDU uses text-based User Datagram Protocol (UDP) communication [3]. These UAV protocols enable effective communication on a given platform, but they are not intended to interoperate semantically across platforms. This implies that custom translation logic or protocol-specific modules are required when integrating different UAV systems [14], thereby increasing system complexity and reducing scalability in multi-drone environments.

### 2.2. Robotics Middleware

Distributed system integration is enabled by middleware frameworks such as the Data Distribution Service (DDS) and the Robotic Operating System (ROS 2), which use publish-subscribe communication paradigms. These frameworks improve adaptability while separating the data sender and receiver. On the other hand, they primarily address the communication layer and do not provide a standard message format for representing UAV information semantically across different systems. Because of these, platform-specific translation methods are necessary for compatibility. For example, integrating ArduPilot (MAVLink), Crazyflie (CRTP), and Tello EDU (UDP-based SDK) drones into a single ROS 2 environment is difficult because each protocol and message requires its own translation tool. The increasing use of various systems significantly complicates integration. Therefore, middleware solutions alone are not sufficient to make different UAV systems semantically interoperable or compatible with one another.

### 2.3. Information Exchange Models

To support data interoperability, several information exchange systems have been created. People from different domains, such as defense and air transportation, share structured and verified information using the National Information Exchange Model (NIEM), which is built on XML and XSD [15,16,17,18]. Other domains, such as biodiversity, the maritime, and autonomous transport industry, also establish data standards for information exchange and quality [19,20,21,22]. However, its application is difficult to understand and isn’t suitable for quick communication between UAVs or for sharing real-time telemetry. Similarly, aircraft standards such as the Flight Information Exchange Model [23] and the Aeronautical Information Exchange Model (AIXM) are more concerned with flight planning and managing aeronautical information than with enabling different UAVs to work together [24]. Military standards such as STANAG 4586 provide UAVs with standardized ways to structure their data and communicate with one another. While they work well for military activities, their complexity makes them hard to use in civilian, research, and educational UAV environments [25].

Existing UAV communication protocols enable efficient platform-level communication but lack unified semantic structures, whereas middleware frameworks require platform-specific translation mechanisms. While STANAG 4586 and NIEM models both provide mature XML/XSD-based interoperability, they are designed for large-scale, domain-specific contexts: NATO military UAS control and broad interagency government information exchange, respectively, and their full specifications entail substantial implementation and schema overhead. Neither is tailored to the lightweight, real-time exchange requirements of small heterogeneous commercial drones that use protocols such as MAVLink, CRTP, and the Tello SDK.

Therefore, a gap remains in the development of a lightweight, UAV-specific interoperability model that abstracts platform differences, supports structured semantic representation through schema validation, and facilitates information exchange across heterogeneous UAV systems, as shown in Table 1. The proposed NDIEM model addresses this gap [26]. Interoperability models commonly use XML, but lighter formats exist with some of the trade-off constraints.JavaScript Object Notation (JSON) [27] is less verbose, but it requires its own schema specification for validation. Similarly, Concise Binary Object Representation (CBOR) is binary and compact, but not human-readable [28]. Protocol Buffers offer efficient encoding at the cost of code generation, and the sharing of schema at both endpoints [29]. Therefore, XML is selected as it directly supports the structural validation. Based on the identified interoperability gap, the following research questions (RQs) are formulated:

- **RQ1:** How much degree of heterogeneous UAV platforms’ information exchange is possible through the proposed NDIEM structure without modifying their native protocols?
- **RQ2:** Does the proposed model maintain a valid schema-compliant message exchange across multiple UAV platforms?
- **RQ3:** What processing overhead is introduced by XML-based semantic normalization in the proposed model?

## 3. Overview of Research Method

**Figure 1:**
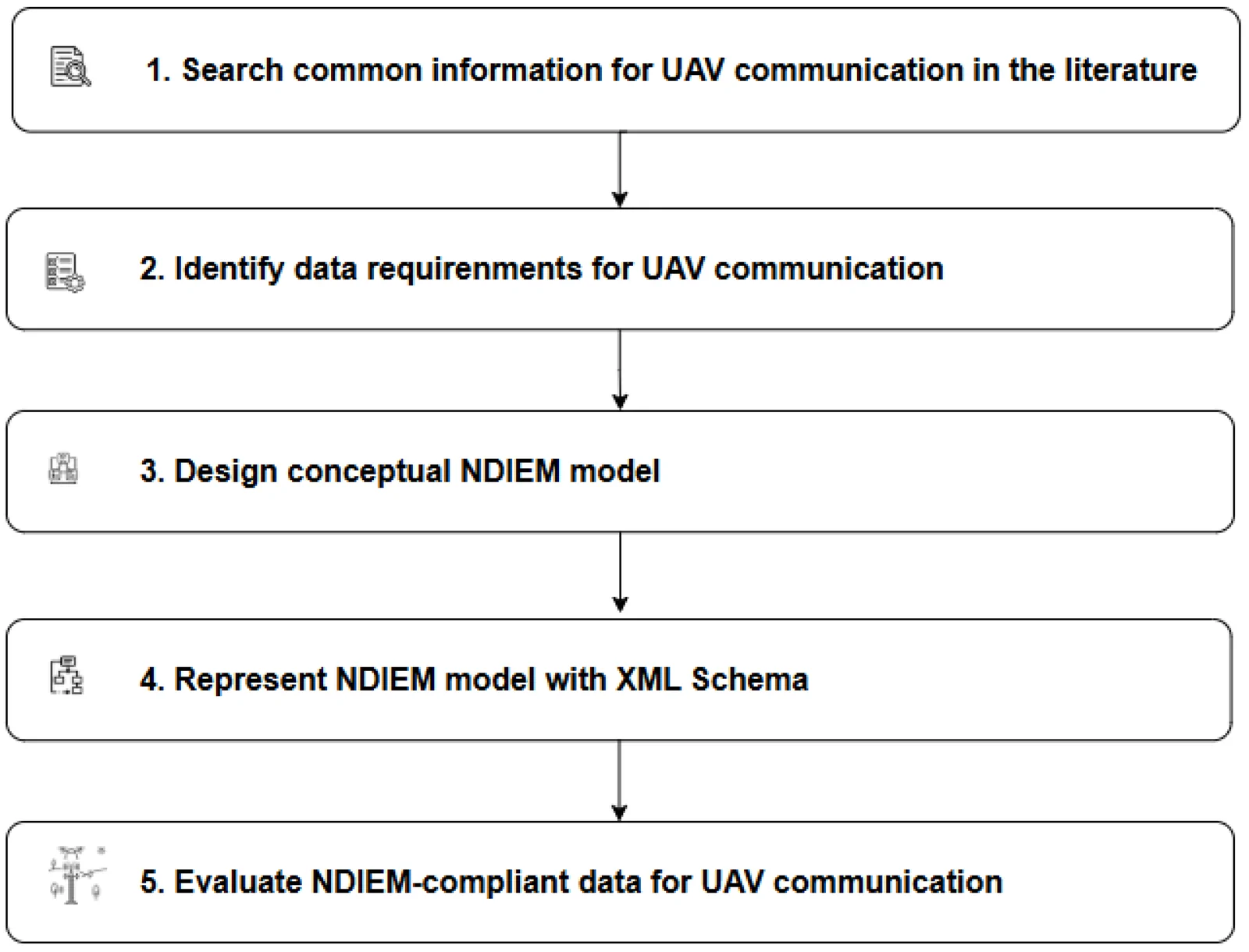
Overview of the research flow.

The development of the proposed NDIEM followed a structured literature-driven research methodology to identify the most recurring and functionally significant information categories used in UAV communication systems. The objective of this process was to ensure that the proposed NDIEM model and data requirements were derived from existing UAV communication and interoperability studies rather than being arbitrarily defined [30]. Figure 1 illustrates the overall research flow adopted in this study.

### 3.1. Search Common Information for UAV Communication in Literature

We conducted a focused literature review to identify studies on UAV communication systems, interoperability, telemetry exchange, autonomous coordination, and the UAV Information exchange model [31,32,33,34]. To achieve comprehensive academic coverage and ensure research reliability, scientific databases such as IEEE Xplore, Scopus, Web of Science, and ScienceDirect were used. The literature search primarily focused on studies published between 2015 and 2025, using keywords related to UAV communication, data exchange, telemetry, sensor information, command and control, and mission data. Duplicate and irrelevant studies were excluded after screening titles, abstracts, and full texts. Finally, the 12 most relevant studies were selected for a detailed analysis of UAV communication and information-exchange structures. The selected studies were analyzed for common information used in UAV communication frameworks and interoperability architectures. The analysis identified five dominant elements that are consistently found in the literature [35]. These include payload/mission data, sensor data, command-and-control data, telemetry data, and drone identification data. This list of elements is used as the basis for the proposed NDIEM model. Based on UML modeling principles and XML-based structural ideas inspired by NIEM methods [36], the identified categories were used to create a conceptual information model.

### 3.2. Identify Data Requirements

After reviewing the literature, a more focused analysis of existing UAV communication protocols and information exchange standards was conducted to determine the basic requirements for the data elements for the proposed NDIEM Model. After the analysis, we found that for drone communication systems to work well, UAVs need to be able to send and receive structured, interoperable data covering identity, command and control, sensor data, telemetry, payload information, and environmental context [37].

#### 3.2.1. Identify Key Data Requirements

The proposed model establishes fundamental data elements for the standardized representation of drone-generated data and facilitates consistent communication between heterogeneous UAV platforms and ground control systems. The importance of message formats, extensibility, and platform independence is also being highlighted in literature and has been taken into account in the design of modular data elements in NDIEM. As a result, the data requirements directly influenced the creation of the core NDIEM components. These data requirements ensured that the model met existing UAV communication needs and enhanced connectivity across different systems [38]. Some of the data requirements for the model are described in Table 2 below.

#### 3.2.2. Identify Structural Requirements

To ensure that NDIEM is effective, it must not only represent standardized UAV information but also maintain structural consistency and enable reliable information exchange across multiple platforms. While the Data Requirements define the core UAV information categories, the structural requirements describe how the information is organized, validated, and exchanged within heterogeneous environments [39]. In multi-UAV scenarios involving different drones, such as Crazyflie, ArduPilot, and Tello EDU, the model must support efficient XML-based representation, cross-platform interoperability, extensible schema organization, and hierarchical structural consistency [40]. These properties are important for ensuring reliable and consistent information exchange during real-time UAV operations. Table 3 summarizes the structural requirements of the proposed NDIEM.

The proposed NDIEM demonstrates a logical relationship between the identified data requirements and the structural requirements [41,42]. To meet the structural requirements, we adopted XML representation, which supports hierarchical and extensible message structure. The remaining processes, Step 3: Design a conceptual model of NDIEM, Step 4: Represent the NDIEM model with XML Schema, and Step 5: Evaluate NDIEM-compliant data for UAV communication in Figure 1, are described in the following sections.

## 4. Proposed NDIEM Information Model

### 4.1. Designing a Conceptual Model of NDIEM

**Figure 2:**
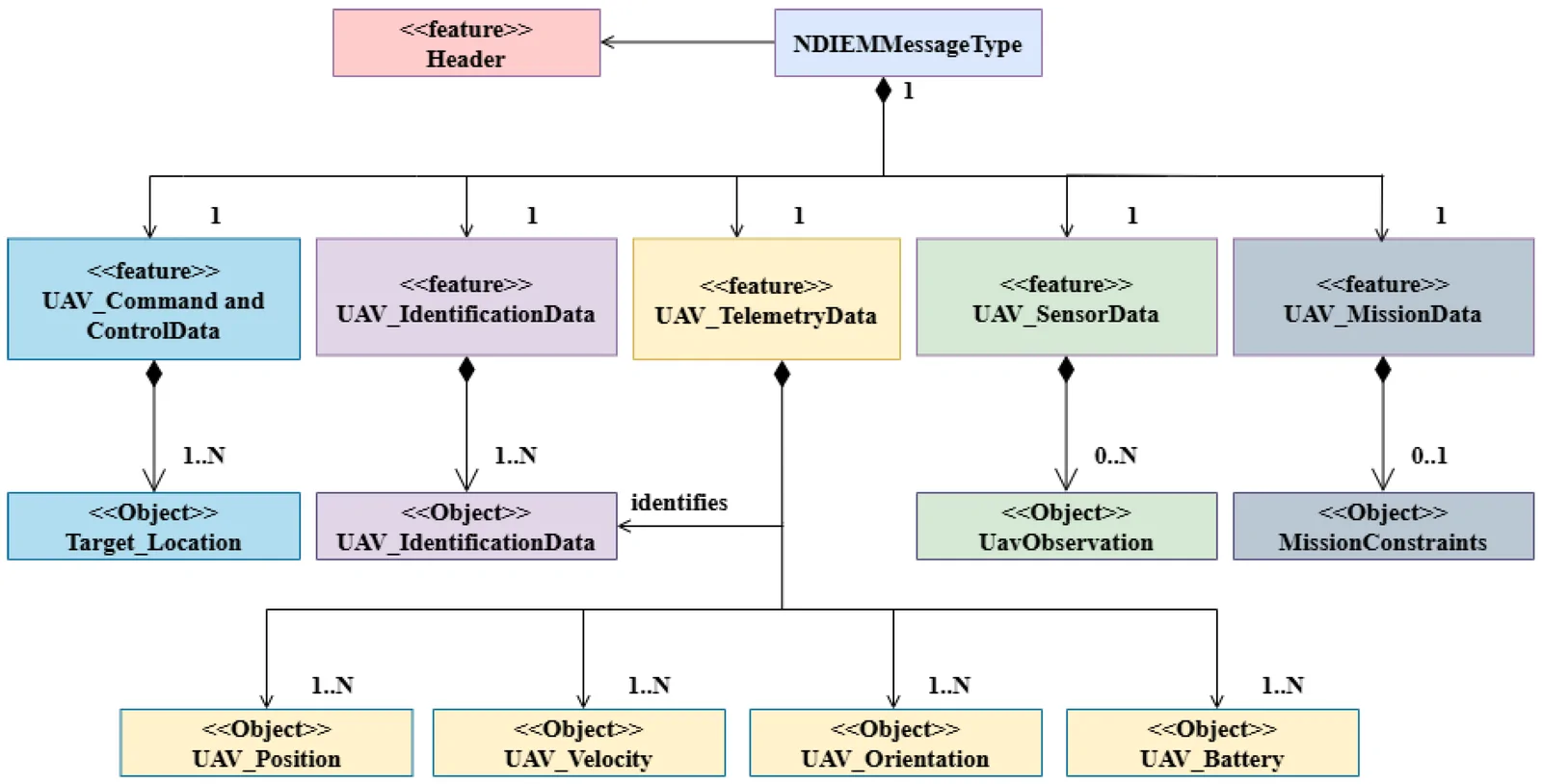
Hierarchy of elements for the NDIEM.

The NDIEM is designed to formalize the complex concepts inherent in multi-UAV operations by organizing information into a structured collection of five primary entities, their associated attributes, and the logical relationships among them. To visualize this data message structure, the NDIEM uses a comprehensive UML class diagram that defines the hierarchical decomposition of the information required for seamless interoperability across heterogeneous platforms. As illustrated in the UML class diagram in Figure 2, the complete drone message structure is anchored by the <NDIEMMessage> root element, which is defined by <NDIEMMessageType> and provides metadata, including a timestamp and messageID.

The <NDIEMMessageType> includes a <Header> that contains the receiverID and senderID. These components ensure that each data item is globally unique and time-synchronized across the heterogeneous fleet, satisfying the basic condition for platform-independent data flow. The high-level structure is further divided into five main key elements, each with a specific operational role. <UAV_IdentificationData> stores immutable platform-specific metadata, such as <uavManufacturer> and <firmwareVersion>, which enables the central NDIEM message to identify the hardware capabilities and software configurations of heterogeneous UAV platforms.

<UAV_TelemetryData> represents the real-time operational state of a UAV, including position, velocity, orientation, and battery status. The model employs strong composition (represented by the black diamond in the UML class diagram) to ensure that essential status objects, such as <UAV_Position> and <UAV_Battery>, remain structurally bound to their parent telemetry object, thereby preventing data fragmentation and preserving message consistency during high-speed data transmission.

<UAV_CommandControlData> enables two-way communication for mission commands, command status, and <Target_Location> coordinates to support accurate navigation and emergency response instructions. <UAV_SensorData> associates raw media assets with corresponding <UAV_Observation> metadata, including camera imagery and LiDAR measurements, thereby standardizing heterogeneous sensor data streams and linking observations to specific geographic locations [43]. Finally, <UAV_MissionData> defines mission constraints, such as <minAltitude> and <maxSpeed>, which serve as safety parameters, operational objectives, and flight plan constraints. NDIEM employs Papyrus Ecore data types (e.g., EDouble for geographic coordinates and EBigDecimal for battery-related metrics) to establish a platform-independent information model capable of satisfying the core data requirements of modern collaborative UAV missions [44].

### 4.2. Representing the NDIEM with XML Schema

NDIEM employs a UML class diagram as its basic model, which is hierarchical and self-describing and therefore important for interoperability in heterogeneous environments. The NDIEM can be modeled with a strict XML Schema Definition (XSD) as an operational contract to the drone’s adapter to ensure the integrity of these exchanges. This scheme helps enforce consistency across all data messages through precise type definitions, cardinality constraints, and semantic rules. The NDIEM was developed using the Eclipse Modeling Framework (EMF) and the Papyrus modeling tool [45], an Eclipse-based framework that enables the construction of standardized and rigorous UML representations.

**Figure 3:**
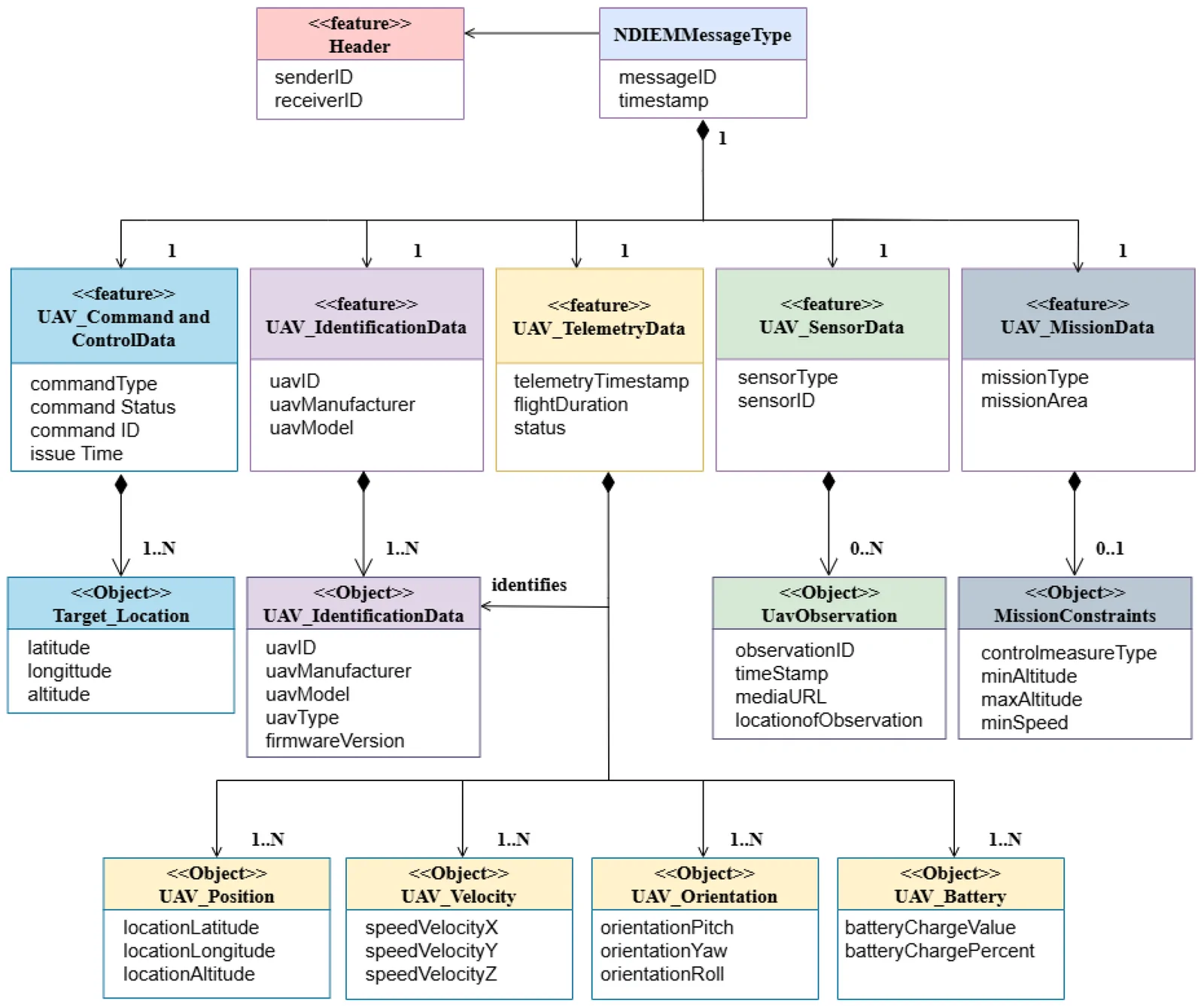
A detailed drone information exchange model represented with a class diagram.

This modeling approach allows for a formal definition of the drone data message structure, as shown in the UML class diagram in Figure 3. All classes, attributes, and their logical relationships are captured accurately. Essential features of these tools include standardized Ecore data types, which are mandatory for maintaining platform independence and high numerical precision across different UAV systems. The model explicitly uses several Ecore types to define its attributes, such as <EString> for identification metadata, categorical fields <EDouble> for high-precision coordinates and velocity vectors, <EInt> for discrete status values, and <EBigDecimal> for sensitive power metrics such as battery charge values [46].

XML’s hierarchical and self-descriptive properties are considered essential to achieving interoperability in heterogeneous environments. These exchanges are validated by Python 3.13.3 and lxml 6.1.1 library, which supports processing XML data. The module verifies three levels: structural (correct nesting), type (numeric format verification), and semantic (enforcing operational constraints). This comprehensive process ensures schema compliance during telemetry operations, providing a validated, scalable solution for multi-drone coordination [47].

The <UAV_IdentificationData> class contains identification-specific attributes, including UAV identifiers, manufacturer details, firmware information, and operational status. The <UAV_TelemetryData> class maintains composition relationships with subordinate classes, including <UAV_Position>, <UAV_Velocity>, <UAV_Orientation>, and <UAV_Battery>, ensuring that all telemetry-related parameters remain structurally grouped within a unified operational context. Similarly, the <UAV_CommandControlData> class encapsulates command status, target coordinates, and navigation directives, while <UAV_SensorData> aggregates sensor observations and media references through nested relationships. The <UAV_MissionData> class further associates mission objectives, waypoint sequences, and operational constraints within a hierarchical structure. These UML-defined class relationships are directly translated into nested XML Schema elements, enabling a consistent, extensible, and interoperable representation of heterogeneous UAV data [48].

**Message Format**: With the individual class objects and attribute elements, the combination of these messages can now be illustrated. The following XML document, Listing 1, represents a single, self-contained message generated by a drone. Listing 1 provides a partial XML schema definition, representing a single standardized NDIEM message type. The complete XML schema (v1.0.0) can be viewed in the NDIEM schema on GitHub [42].

**Listing 1.** Partial XML Schema Definition of the Proposed NDIEM Message Structure.
<?xml version="1.0" encoding="UTF-8"?>

<xs:schema xmlns:xs="http://www.w3.org/2001/XMLSchema"

           targetNamespace="http://finalNdiem/1.0"

           xmlns:ndiem="http://finalNdiem/1.0"

           elementFormDefault="qualified">

  <xs:element name="NDIEMMessage" type="ndiem:NDIEMMessageType"/>

  <xs:complexType name="NDIEMMessageType">

    <xs:sequence>

      <xs:element name="messageID"type="xs:string"/>

      <xs:element name="timestamp"type="xs:dateTime"/>

      <xs:element name="UavIdentificationData" type="ndiem:UavIdentificationDataType"/>

      <xs:element name="UavCommandAndControlData"

        type="ndiem:UavCommandAndControl

        DataType"minOccurs="0"/>

    </xs:sequence>

  </xs:complexType>

  <xs:complexType name="HeaderType">

    <xs:sequence>

      <xs:element name="senderID" type="xs:string"/>

      <xs:element name="receiverID" type="xs:string"/>

    </xs:sequence>

  </xs:complexType>

</xs:schema>


**NDIEM Key Data Elements:** The NDIEM information exchange model is organized into five key data elements: <UAV_IdentificationData>, <UAV_TelemetryData>, <UAV_SensorData>, <UAV_CommandControlData>, and <UAV_MissionData>. Each element is designed to address a specific operational requirement for collaborative UAV missions. Detailed descriptions of these elements are provided in the Appendix A.

## 5. NDIEM in Action

This section demonstrates the proposed NDIEM framework through a conceptual multi-UAV interoperability scenario and prototype-based experimentation.

### 5.1. Scenario

To evaluate the proposed NDIEM, a controlled, conceptual, hypothetical scenario is defined in which heterogeneous UAV systems are modeled as abstract data-generating and data-consuming entities. The primary objective of this scenario is not to simulate physical flight operations [49] but to evaluate the proposed NDIEM model’s capability to support structured data integration, standardized message processing, and structural interoperability across heterogeneous UAV communication environments [50]. In addition to general heterogeneous UAV communication settings, a wildlife emergency response scenario is introduced to provide a practical conceptual context for the proposed model.

**A Hypothetical Scenario:**

*The conceptual validation scenario considers a coordinated emergency response operation in a forest or wildlife reserve, where multiple agencies collaborate to address a forest fire. The participating response departments include the fire department, police department, medical emergency department, and disaster headquarters coordinated through the Ground Control Station (GCS). The fire department focuses on fire detection, the police department monitors the affected area, and the medical department coordinates medical aid for the emergency. During the emergency operation, each department deploys heterogeneous UAV systems operating under different communication protocols. The UAVs take off and continuously communicate with the GCS, exchanging mission information and telemetry data throughout the operation. The GCS coordinates the deployed UAVs by receiving operational information and distributing standardized information to the corresponding emergency response departments. Based on the information received, fire response units coordinate firefighting operations, police departments monitor the operational area and maintain situational awareness, and medical UAVs are dispatched to rescue injured individuals requiring emergency assistance.*

**Figure 4:**
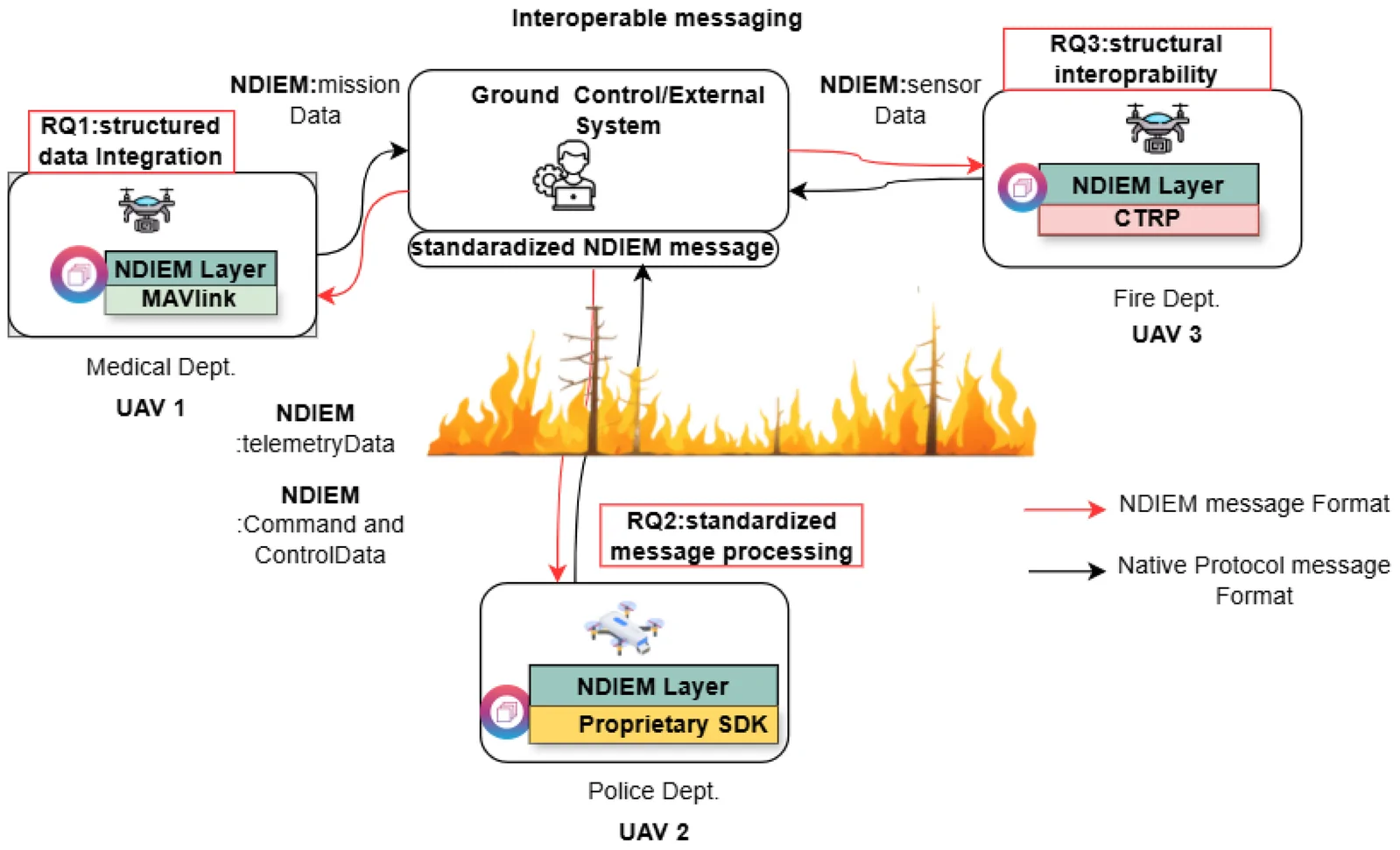
NDIEM multi-UAV wildlife scenario illustration.

As illustrated in Figure 4, the scenario takes place in an emergency response environment in a forest area, where multiple UAV agencies collaboratively exchange information for situational awareness and response coordination. The participating response departments include the fire department, police department, and medical emergency department.

In this scenario, heterogeneous UAV systems operating under different communication protocols are assumed to capture and transmit operational information for environmental monitoring and emergency response. The UAV systems include MAVLink-based UAV platforms, CRTP-based swarm UAV systems, and SDK/API-based educational UAV platforms [51]. The gathered information includes sensor data (fire detection indicators and wildlife movement observations), environmental data (environmental monitoring data), metadata (geospatial metadata), telemetry data (system and communication status information), and command-and-control data (emergency response alerts). GCS serves as a centralized coordination and validation node where standardized NDIEM messages are received, processed, and distributed to various emergency response efforts. Fire response units interpret fire-related structured alerts, police departments access situational awareness and location-based information, and medical teams process emergency-related operational metadata through a unified, structurally consistent representation.

The conceptual scenario, therefore, demonstrates how heterogeneous UAV-generated information can be consistently transformed into a standardized XML-based representation independent of the originating communication protocol. The scenario further illustrates the applicability of the proposed NDIEM model for enabling coordinated multi-domain information interpretation through a unified data representation. No assumptions are made regarding autonomous flight coordination, real-world deployment infrastructure, or physical flight dynamics. The scenario is intentionally maintained at a conceptual and prototype-validation level to evaluate the feasibility of the proposed NDIEM model under controlled heterogeneous communication conditions.

### 5.2. Experimental Environment

**Figure 5:**
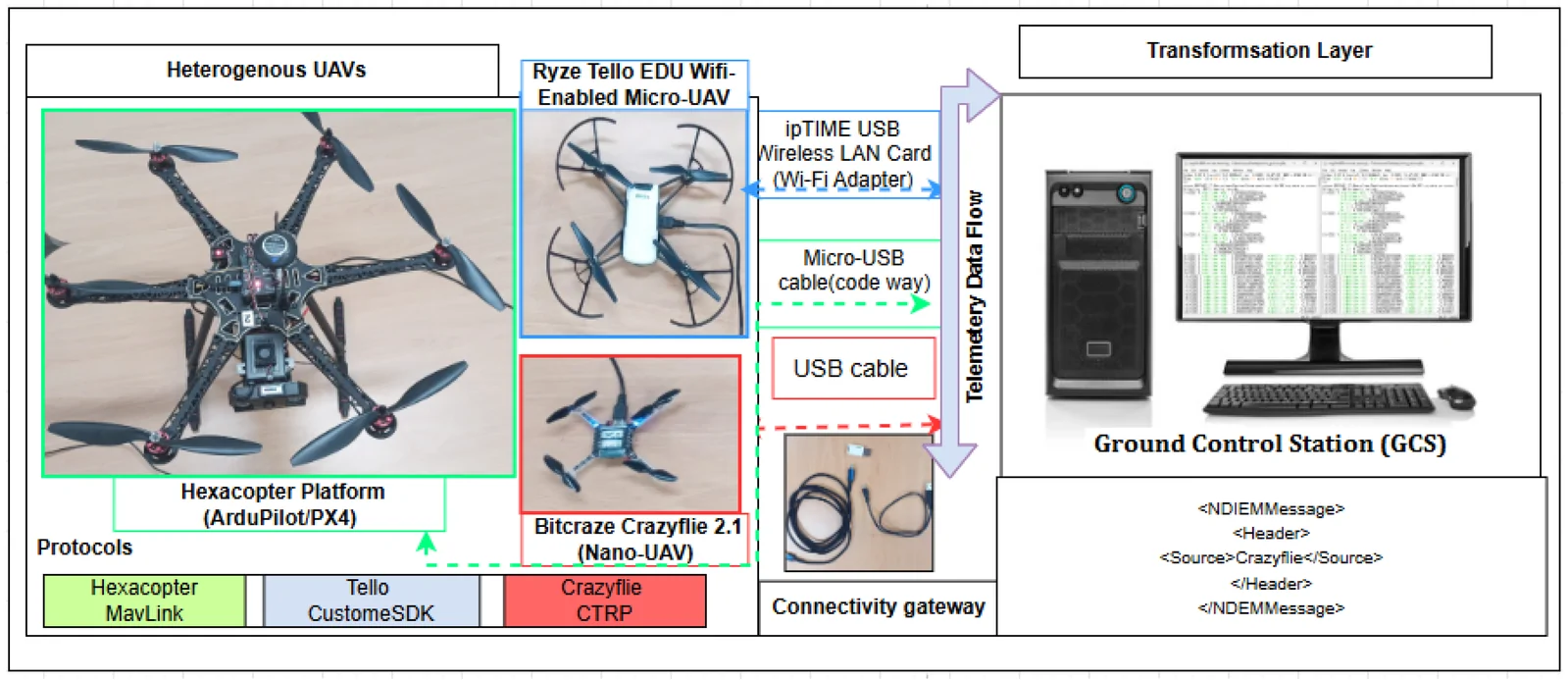
Heterogeneous drones used in the experimentation.

The experimental environment is designed to evaluate the capabilities of the proposed NDIEM under heterogeneous UAV communication conditions. The objective is to assess whether NDIEM can effectively normalize, transform, and validate multi-protocol UAV data into a unified XML-based representation for consistent interpretation at GCS. The experimental environment is constructed using three heterogeneous UAV platforms and their communication protocols, as illustrated in Figure 5. The environment includes the Hexacopter, which uses the MAVLink protocol and represents an industrial-grade UAV system with high-precision telemetry and autonomous flight operations [36]. Crazyflie with the CRTP protocol is a micro-UAV swarm system used in research environments, featuring lightweight, high-frequency telemetry. Tello EDU is an SDK-based educational UAV. It uses UDP-based messaging and custom parsing logic, helping create a heterogeneous environment for testing NDIEM’s adaptability. Each UAV is integrated with an NDIEM-compatible message handler, enabling conversion of native telemetry streams into standardized XML-based NDIEM messages. The GCS serves as the central validation and interpretation unit within multi-agency coordination environments.

### 5.3. From Concept to Prototype System

**Figure 6:**
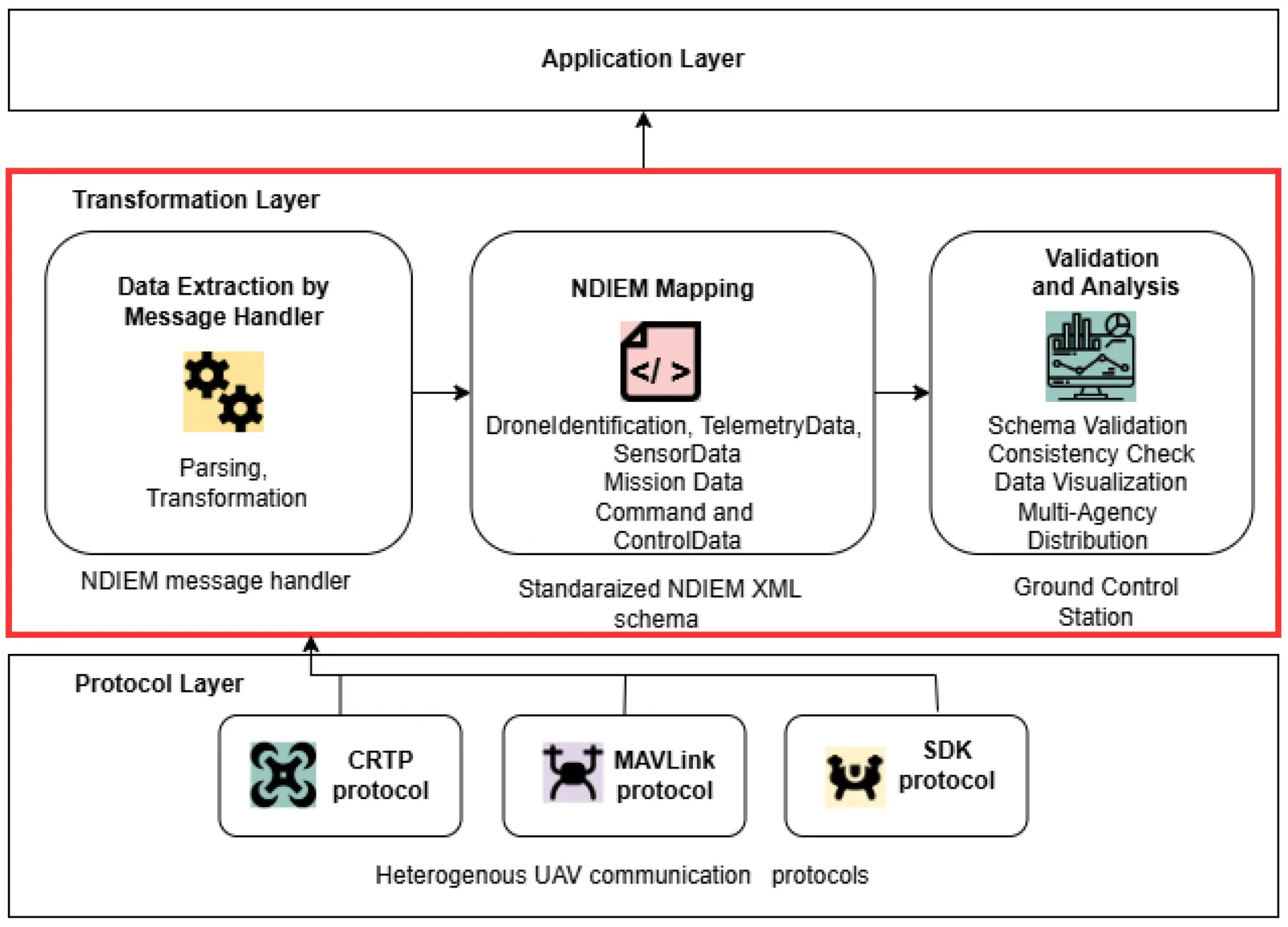
From native UAV protocols to NDIEM message transformation flow.

To operationalize the proposed NDIEM model, a prototype system is implemented to process real-time UAV telemetry data captured directly from onboard sensors, as described in Section 5.5. The prototype system focuses on data transformation and validation logic rather than flight dynamics. Each UAV data source goes through three stages. In our prototype system, the messages are processed in the transformation layer, which communicates with the application layer or lower-level layers, as shown in the red box in Figure 6.

- Data Extraction: Native UAV messages in the protocol layer, e.g., MAVLink, CRTP, or UDP/API, are extracted and parsed.
- NDIEM Mapping: Extracted data are mapped into predefined, standardized NDIEM categories, including Drone Identification, Telemetry Data, Sensor Data, Environmental Data, and Command and Control information.
- XML Serialization: The mapped data are structured into a unified XML schema compliant with NDIEM specifications and then used in application systems.

Incoming XML messages are validated for schema conformance and semantic consistency rules at GCS. The GCS then disseminates interpreted data to the logical endpoints of agencies, enabling the shared UAV information to be interpreted in a domain-specific manner. This prototype thus operates NDIEM as a data interoperability pipeline rather than a physical system deployment. The source code for the prototype system, experimentation procedures, raw telemetry logs, and other useful information are available on GitHub [42].

### 5.4. Ablation Study

To evaluate the pros and cons of the proposed NDIEM model, an ablation study was conducted to assess the contributions of its individual components. In this study, we systematically turn off key modules and measure the impact on performance metrics, including latency, interoperability, validation success, and data integrity (see Table 4).

The evaluation metrics in Table 4 are defined as follows. Here, end-to-end processing time is the total time for a message to traverse the complete pipeline, comprising transformation, XML construction, schema validation, and serialization in the prototype system. Interoperability (%) is the proportion of exchanged messages that the receiver can validate and parse. Data Integrity (%) is the proportion of mapped field values unchanged after the native message format to XML parsing. Validation (%) is the proportion of generated NDIEM messages that are schema-validated. These metrics are selected because they capture the three requirements for structural interoperability, no information is lost during message transformation, and heterogeneous UAVs can consume them.

All ablation metrics were computed over the 2482 NDIEM-mappable messages captured from the three physical drones described in Section 5.5 (1000 from the Crazyflie, 1000 from the Tello EDU, and 1000 from the Hexacopter, out of which 482 telemetry-bearing messages), comprising 12,585 individual field values. The field-value count corresponds to the total number of populated XML elements across all transformed NDIEM messages after native-field mapping. As different UAV platforms expose different telemetry attributes, the number of populated fields varies between messages.

Interoperability and validation are measured per message, while data integrity is measured per field value. Each configuration was executed across 10 independent runs, and the reported values are means, with raw counts in parentheses. N/A indicates that the metric is not applicable because the corresponding component was removed from the evaluated configuration.

**Figure 7:**
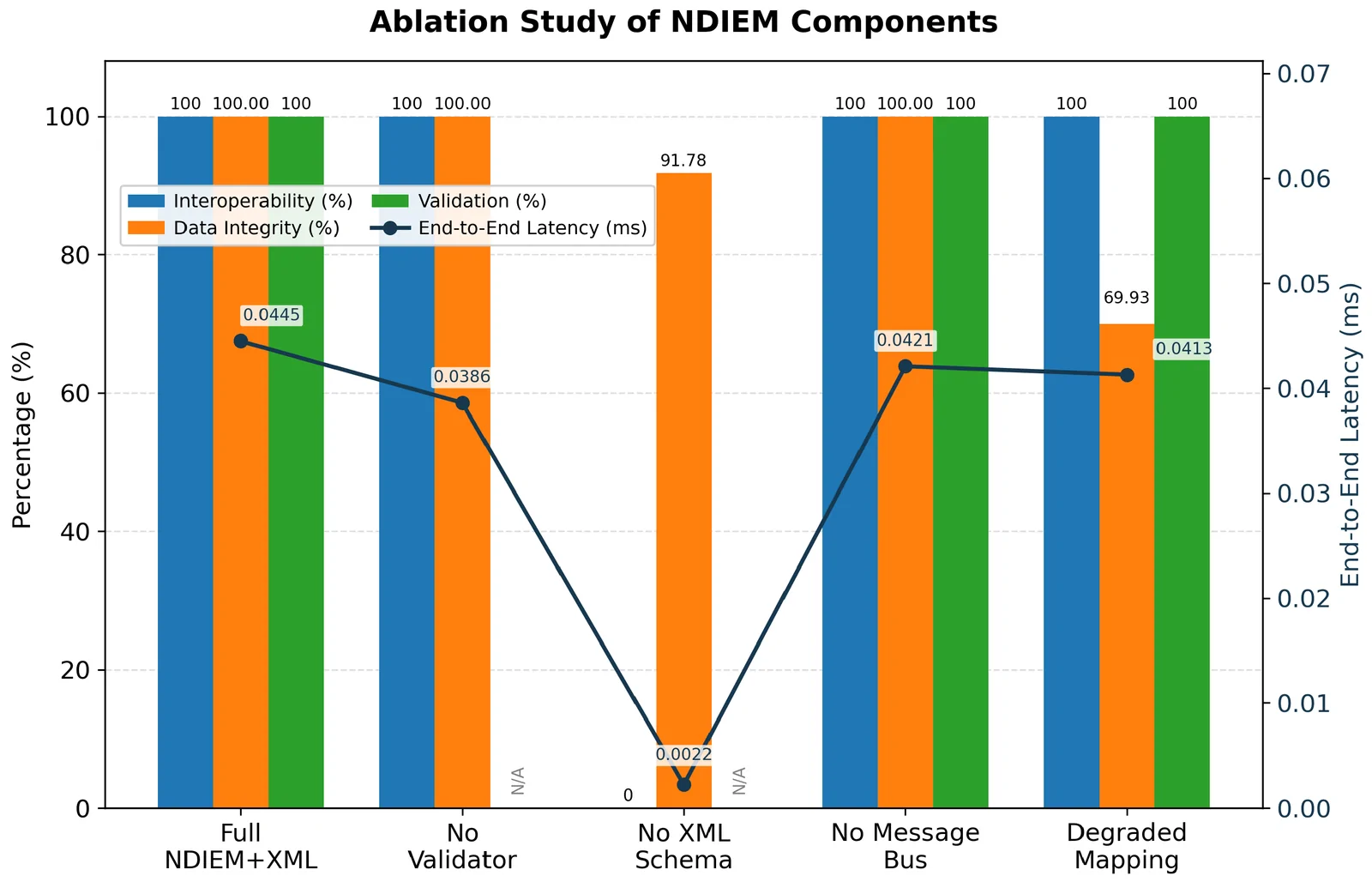
Ablation study results for the proposed NDIEM.

Removing the XML schema representation in XML builder from the communication message reduced interoperability from 100% to 0%, confirming that structured representation is essential for cross-platform understanding. Although 91.78% of data values were preserved, the receiver side could not correctly interpret telemetry fields without a common schema structure. Removing the validator slightly reduced processing time from 0.0445 ms to 0.0386 ms without affecting interoperability or data integrity, but eliminated the ability to verify message conformity. Degraded semantic mapping, map the values incorrectly in the NDIEM message, reduced data integrity to 69.93%, demonstrating the importance of accurate mapping rules for preserving telemetry meaning. Removing the message bus had minimal effect on processing time (from 0.0445 ms to 0.0421 ms) without affecting interoperability, as shown in Figure 7. As discussed above, the message bus contributes to architectural-level design by enabling decoupling and scalability rather than reducing latency.

Overall, these results show that protocol adapters, XML schema representation, and the validation step are the components most responsible for interoperability and data integrity. The complete configuration achieves the best structural interoperability, integrity, and validation performance, while removing the schema or validation markedly reduces interoperability and consistency. Latency remains low across all configurations (0.0022–0.0445 ms, a maximum variation of 0.0423 ms), indicating that these interoperability gains are obtained with minimal processing overhead.

### 5.5. Experimental Results

The experimental evaluation captures real telemetry from the onboard sensors of three physical drones: Crazyflie 2.1 (CRTP) via the cflib Python library, a Tello EDU via native Tello SDK over UDP, and an ArduPilot-based Hexacopter (MAVLink) via Pymavlink. Each platform was stationary but manually repositioned/handled to induce realistic variation in orientation, position, and sensor readings. A Python-based capture script interfaces with each drone’s native protocol to log this telemetry in real time, which is then passed through the NDIEM transformation layer.

The experimental results in Table 5 and Table 6 demonstrate that the NDIEM transformation layer introduces negligible computational overhead, with sub-millisecond processing latency observed across UAV pipelines. The Tello EDU pipeline exhibits slightly higher latency due to larger message payloads. In contrast, the Crazyflie stream shows marginally lower transformation time due to simpler data structures. Each NDIEM message is validated against the XSD schema at generation time on the sending side, immediately before transmission, and again on the receiving side upon reception, before parsing. Furthermore, interoperability between Crazyflie and Hexacopter is validated through message exchange, directly addressing the RQ1-RQ3 evaluation objectives.

#### 5.5.1. Schema Transformation and Field Completeness

Table 5 shows the schema transformation rate and the field completeness across all the tested drones. Data completeness ranges from 27.3% to 100% and depends on the protocol, reflecting structural differences between lightweight protocols such as CRTP/UDP and feature-rich protocols such as MAVLink. However, for the NDIEM representation, 69.7% indicated that the transformation of the fields captures about half of the telemetry elements from the telemetry stream data and handles protocol-specific variations. All telemetry data streams with common telemetry data were successfully transformed into NDIEM format with zero failures or data corruption, as indicated with ✓. The field of Data Completeness measures the percentage of NDIEM telemetry fields that a UAV can fill out of all available fields. A UAV with fewer sensors naturally fills fewer fields.

#### 5.5.2. Transformation Latency and Message Processing

**Figure 8:**
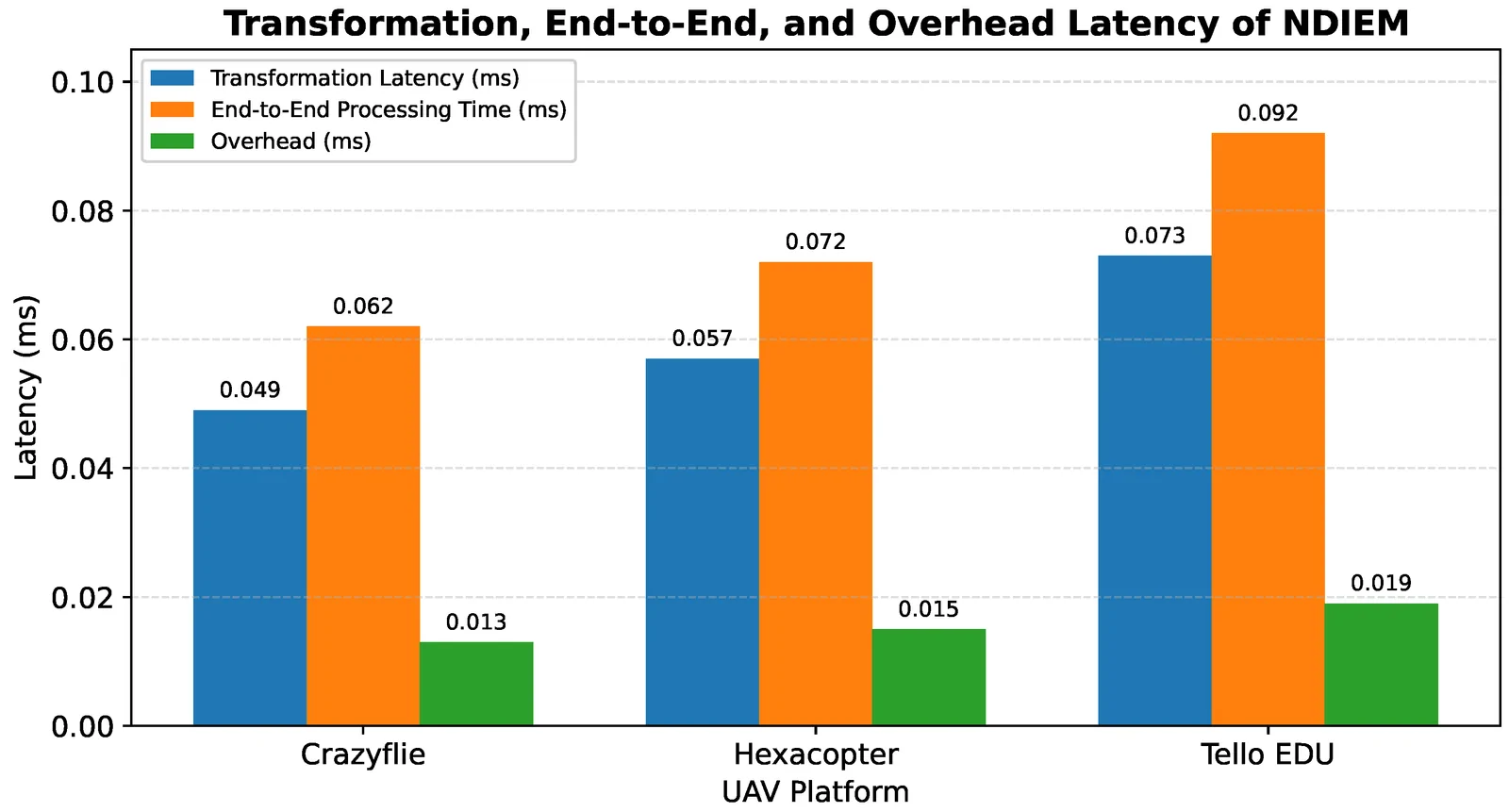
Transformation, end-to-end, and overhead latency.

Table 6 shows that NDIEM introduces a small and consistent processing overhead across heterogeneous UAV platforms. Transformation latency is the execution time required by the Message Handler Layer to transform an extracted native UAV message into its NDIEM XML representation. It measures only the transformation stage and excludes message acquisition, schema validation, and serialization. The transformation latency ranges from 0.049 ms for Crazyflie to 0.073 ms for Tello EDU, while the complete end-to-end processing time remains below 0.1 ms for all evaluated platforms. The additional overhead is mainly attributed to protocol parsing, XML serialization, schema validation, and semantic mapping operations required for interoperability. These results demonstrate that NDIEM achieves efficient cross-platform data transformation while maintaining near real-time processing capability. Figure 8 presents the transformation success rate, field completeness, and average processing latency after protocol-to-NDIEM conversion.

The experimental results demonstrate 100% successful transformation across all platforms while maintaining low overhead and supporting heterogeneous telemetry structures.

#### 5.5.3. Interoperability and Protocol Bridging

**Figure 9:**
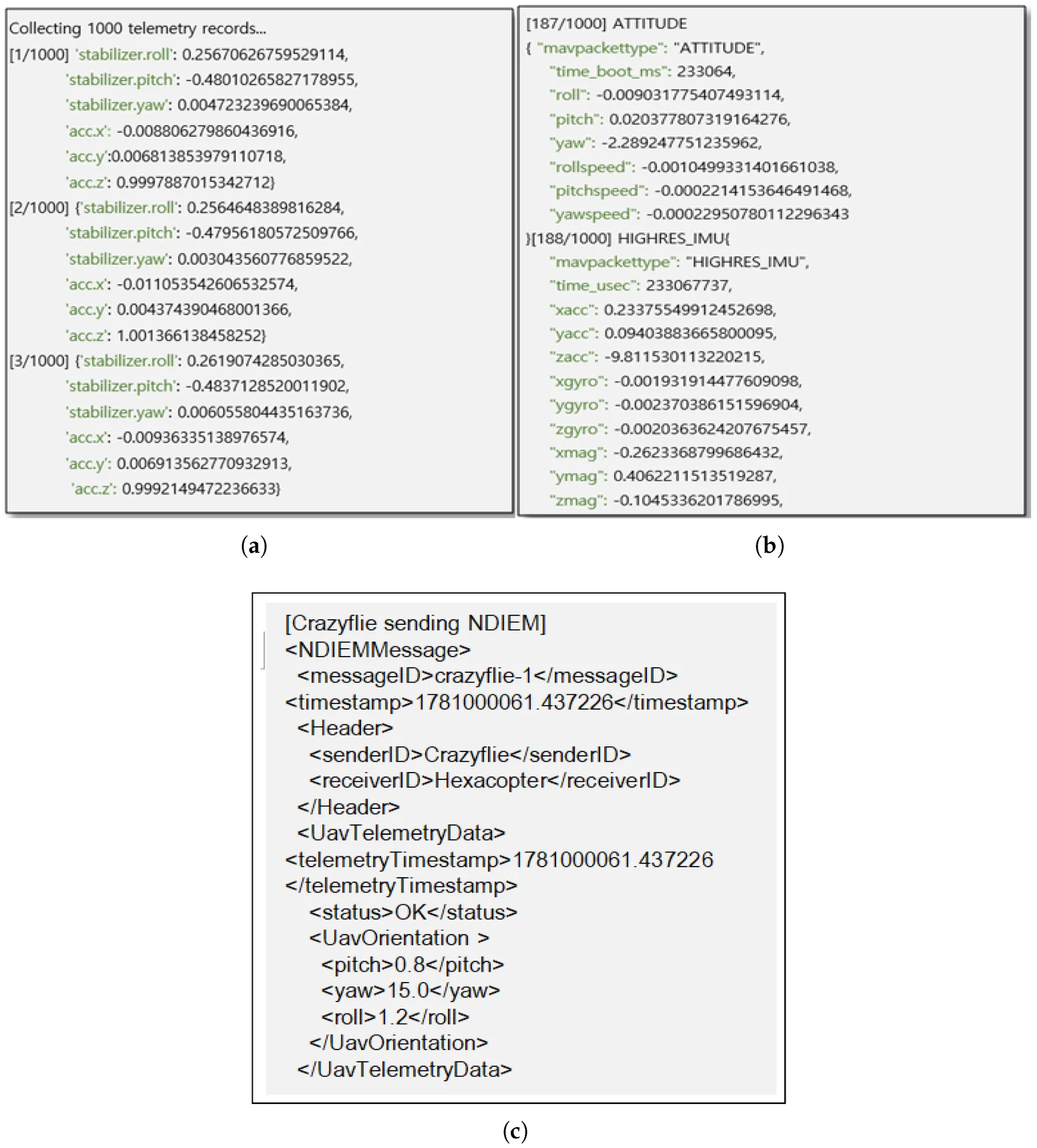
Experimental telemetry acquisition and transformation results: (**a**) terminal output from Crazyflie 2.1, (**b**) raw telemetry data from the Hexacopter, and (**c**) mapping the raw messages into the NDIEM message format.

Interoperability is achieved through a shared NDIEM schema, which enables a Crazyflie UAV to transmit structured telemetry data to a Hexacopter without requiring protocol-level compatibility between the two drones, as shown in Figure 9.

This validates NDIEM using real, sensor-generated telemetry from three physical UAV platforms under static, non-flight conditions, where sensor variation was manually induced. Full in-flight validation, including dynamic multi-UAV coordination and real-time GCS interoperability, is left for future work.

## 6. Discussion

The experimental results show that NDIEM effectively addresses key interoperability challenges in a heterogeneous UAV environment. The model ensures structural reliability and consistency across all messages exchanged through semantic normalization and XSD validation. NDIEM supports the integration of heterogeneous UAV platforms through an XML-based structural message exchange, without requiring modification of existing native communication protocols.

In the illustrated scenario, NDIEM messages are structured hierarchically, facilitating semantic interoperability. NDIEM structures UAV data into five key element categories: identification, telemetry, command and control, sensor, and mission data. This kind of structured, modular categorization guarantees the completeness of the information representation and high extensibility. Additional data elements such as extended sensor modalities, AI-driven analytics, or payload-specific attributes can be added without re-engineering the core schema. Further protocol-specific adapters, such as MAVLink, CRTP, and the Tello EDU SDK, can support bidirectional translation between native protocols and the canonical NDIEM XML structure. The model is compatible with conventional requirements-based systems as well as modern IP-based (TCP/IP) architectures, greatly increasing deployment flexibility across different operational environments.

An important contribution to this work is the identification of key elements for information exchange among the different UAVs. Moreover, in our work, we use a Python-based message handler compatible with NDIEM, implemented at the UAV and GCS levels. It provides protocol abstraction, making communication independent of the underlying transport mechanisms. The benefit of XML in interoperability, extensibility, maintainability, and unified monitoring far outweighs the slight parsing overhead compared to the lightweight binary protocols in heterogeneous multi-UAV coordination scenarios. The model supports selective data processing and filtering at the adapter layer, which gives it strong scalability potential for operations with higher swarm density. It defines a common, protocol-agnostic schema that maps each platform’s native format, so that heterogeneous drones operating on different protocols can exchange structurally consistent information regardless of the underlying transport.

### Threats to Validity and Limitations

The results are promising, but several limitations should be pointed out. The evaluation was performed on only three UAV platforms in a controlled lab environment and was not tested in large-scale outdoor deployments under dynamic network conditions and interference. This represents an external validity threat, as the findings may not fully generalize to diverse operational environments, larger heterogeneous UAVs, different communication infrastructures, or mission-critical deployment scenarios. We plan to apply NDIEM to diverse UAV platforms and under different communication protocols.

To further confirm the scalability and resilience of the proposed model, more experimental work involving heterogeneous UAV swarms, field trials outside, long-range communication links, and varied ambient conditions is required. In addition, the XML-based representation increases message size and processing overhead compared with optimized binary formats, potentially requiring additional optimization for very high-frequency swarm telemetry. The available adapters in the prototype system only implement MAVLink, CRTP, and Tello EDU SDK protocols.

Security is an important quality requirement in communication. The current model favors structural and partial semantic interoperability over security features such as encryption and authentication. This provides internal validity, as the absence of robust security measures could affect how the model behaves and performs in practical operating scenarios requiring secure communication.

Although NDIEM is consistent with the general principles of standardization, it has some limitations. NDIEM does not yet conform to implement mappings with established international Unmanned Aircraft System (UAS) standards, such as the International Organization for Standardization (ISO) 21384 series for UAS operational definitions, ASTM International Committee F38 standards for UAS interoperability principles, the NATO Standardization Agreement (STANAG) 4586 for unmanned aerial vehicle control system interoperability, or the Open Geospatial Consortium (OGC) Sensor Things API for sensor and IoT data exchange. Standardization alignment remains future work, particularly to support regulatory adoption and broader operational deployment.

## 7. Conclusions and Future Work

In this study, we presented an information exchange model, NDIEM, to support interoperability among heterogeneous UAV platforms. It provides standard XML-based communication that can organize the UAV’s information into Identification, Telemetry, Command and Control, Sensor, and Mission categories. The model enables standardized information exchange across heterogeneous UAV communication protocols, such as MAVLink, CRTP, and UDP/API-based systems, through protocol-specific adapters and prototype-based validation. The experimental results demonstrate the feasibility of interoperability and real-time message processing in representative communication scenarios involving heterogeneous UAVs, using the conceptual integration and prototype. The experimental evaluation demonstrates that the model enables standard message organization, validation, and cross-platform interpretation while preserving stable operational behavior during coordinated mission execution. This model decouples native communication protocols from standardized mechanisms for information exchange, providing a foundation for scalable and extensible research into UAVs’ structural interoperability.

Future research should examine how to integrate access control, encryption, authentication, and resilience to cyber threats, while assessing their effects on interoperability performance. It should also explore other serialization formats, such as Concise Binary Object Representation (CBOR), JSON, and Protocol Buffers, as alternatives to XML for bandwidth-constrained UAV applications.

## Figures and Tables

**Table 1:** Comparison of Existing UAV Data Exchange Approaches with the Proposed Model.

Approach	Format	Semantic Structure	Multi-Platform	UAV Specific
ROS 2/DDS [10]	Various	Platform-dependent	Yes	No
MAVLink [12]	Binary/XML	None	Limited	Yes
CRTP [2]	Binary	None	No (Crazyflie only)	Yes
Tello SDK [3]	None	None	No (Tello only)	Yes
FIXM [23]	XML	Flight planning	Aviation only	No
NIEM [15]	XML/XSD	Domain namespaces	Cross-agency	No
STANAG 4586 [16]	XML/XSD	Functional categories	Military only	Yes
AIXM [17]	XML/GML	Aeronautical information	Aviation only	No
Proposed Model	XML/XSD	Five core data elements	Tested for three UAVs	Yes

**Table 2:** Data Requirements for the NDIEM.

Requirement ID	Requirement Name	Description
**DR1**	UAV Identification Data	The model must provide a standardized structure for representing UAV identification information, including unique drone identifiers, platform type, manufacturer details, and metadata required for interoperable UAV recognition across heterogeneous systems.
**DR2**	Telemetry Data	The model must support a standardized representation of essential telemetry information, including position, three-dimensional velocity, orientation (pitch, roll, and yaw), altitude, and battery status, to enable interoperable data exchange.
**DR3**	Command and Control Data Exchange	The model must represent command-related information, including waypoints, flight modes, emergency commands, and execution status updates in a consistent exchangeable format across heterogeneous UAV systems.
**DR4**	Sensor Data Representation	The model must support a structured representation of sensor observations, including sensor type, observation identifiers, timestamps, environmental measurements, payload information, and associated sensor characteristics.
**DR5**	Mission Data	The model must provide a standardized structure for representing mission-related information, including mission identifiers, mission objectives, operational areas, altitude constraints, speed limitations, mission status, and task execution details.
**DR6**	Metadata Representation	The model must support representing metadata related to the UAV’s key data elements.

**Table 3:** Structural Requirements for the NDIEM.

Requirement ID	Requirement Name	Description
**SR1**	Efficient Structural Representation	The model should define well-structured XML message representations that support efficient parsing, processing, and schema validation during information exchange.
**SR2**	Cross-Platform Use	The model must remain protocol-independent and support normalization of heterogeneous communication formats, including MAVLink, UDP-based SDKs, and custom telemetry streams into a unified XML representation.
**SR3**	Extensible Information Structure	The model should support extensibility by adding new UAV information categories, attributes, and sensor elements while maintaining compatibility with the existing core schema.
**SR4**	Structural Consistency	The model should preserve strong parent-child relationships between UAV information classes to maintain structural consistency and contextual integrity during information exchange.

**Table 4:** Ablation Study Results with NDIEM Message Impact in the Prototype System.

Configuration	End-to-End Processing Time (ms)	Interoperability (%)	Data Integrity (%)	Validation (%)
Baseline (Full NDIEM with XML)	0.0445	100 (2482/2482)	100 (12,585/12,585)	100 (2482/2482)
No Validator	0.0386	100 (2482/2482)	100 (12,585/12,585)	N/A
No XML Builder	0.0022	0 (0/2482)	91.78 (11,550/12,585)	N/A
No Message Bus	0.0421	100 (2482/2482)	100 (12,585/12,585)	100 (2482/2482)
Degraded Mapping	0.0413	100 (2482/2482)	69.93 (8801/12,585)	100 (2482/2482)

**Table 5:** Schema Transformation and Completeness of Telemetry Data Elements.

Native Protocol	Drone	Telemetry Fields	Data Completeness	Field Transformation
CRTP	Crazyflie	Roll, Pitch, Yaw	27.3% (3/11 fields)	✓
SDK (UDP-based)	Tello EDU	Roll, Pitch, Yaw, Vx, Vy, Vz, Alt, chargeValue, chargePercentage	81.8% (9/11 fields)	✓
MAVLink	Hexacopter	Roll, Pitch, Yaw, lat, lon, Vx, Vy, Vz, Alt, chargeValue, chargePercentage	100% (11/11 fields)	✓
Proposed Approach	All three drones	Unified telemetry fields	69.7% (average)	✓

**Table 6:** Transformation Latency and End-to-End Processing Time of the NDIEM Message Processing Pipeline (mean ± standard deviation over 5 runs).

UAV Platform	Transformation Latency (ms)	End-to-End Processing Time (ms)	Overhead (ms)
Crazyflie	0.049±0.001	0.062±0.001	0.013
Hexacopter	0.057±0.011	0.072±0.014	0.015
Tello EDU	0.073±0.001	0.092±0.002	0.019

## Data Availability

The raw telemetry logs, NDIEM XSD schema, and capture scripts are openly available at https://github.com/B-ushra/NDIEM-A-Networked-Drone-Information-Exchange-Model-for-Heterogenous-Drones (accessed on 5 August 2026).

## DURC Statement

The current research is limited to conceptual modeling in software engineering and drone development, which is beneficial for exchanging data among drones as a proof of concept and does not pose a threat to public health or national security. The authors acknowledge the dual-use potential of the research involving the data exchange model in drone software development and confirm that all necessary precautions have been taken to prevent potential misuse. As an ethical responsibility, the authors strictly adhere to relevant national and international laws about DURC. The authors advocate for responsible deployment, ethical considerations, regulatory compliance, and transparent reporting to mitigate misuse risks and foster beneficial outcomes.

## Appendix A

To facilitate a comprehensive understanding of the proposed NDIEM, Appendix A provides detailed descriptions of the five key data elements. These elements represent the fundamental components of the information model and serve as the basis for UAV data representation, exchange, and interoperability. The detailed structure and associated attributes of each element are presented below.

**UAV Identification Data:** In NDIEM, the <UAV_IdentificationData> package acts as the “digital identity card” for each asset involved in heterogeneous UAV coordination. The detailed data elements of the UAV identification type are presented in Table A1.

**Table A1:** Specification for the UAV_IdentificationData.

Attribute	Data Type	Description
uavID	EString	A globally unique machine-readable identifier assigned to each UAV platform. This ensures that data originating from heterogeneous platforms such as PX4, ArduPilot, Crazyflie, or Tello EDU systems can be uniquely distinguished during collaborative operations.
UavManufacturer	EString	Identifies the UAV platform’s originating manufacturer or ecosystem (e.g., DJI, Bitcraze, Ryze). This attribute helps the system determine the corresponding protocol-specific translation and interoperability requirements.
UavModel	EString	Specifies the exact hardware model of the UAV platform (e.g., Tello EDU, Crazyflie 2.1). This information supports platform-aware mission evaluation and capability analysis.
UavType	EString	Represents the physical UAV configuration, such as a quadcopter or fixed-wing UAV. This classification assists in applying mission-specific operational constraints and compatibility checks.
FirmwareVersion	EString	Indicates the current onboard firmware or software version installed on the UAV platform. This attribute supports compatibility verification between UAV capabilities and the NDIEM schema structure.

**Telemetry Data:** Telemetry data serve as a centralized container for time-sensitive flight metrics. Their primary purpose is to implement unified telemetry by providing a low-latency pathway for critical UAV status updates. The <UAV_TelemetryData> class is defined by three top-level attributes: timestamp, duration, and status. To better understand the operational role and data structure of <UAV_TelemetryData> within the NDIEM framework, the detailed specification is presented in Table A2.

**Table A2:** Specification for the UAV_TelemetryData.

Attribute	Data Type	Description
timestamp	EInt	High-precision integer used for temporal synchronization of telemetry updates, enabling accurate reconstruction of flight paths and event ordering.
flightDuration	EInt	An optional field representing elapsed mission time, used for mission tracking and performance analysis.
status	EInt	Encodes the UAV’s current operational state, supporting system-level monitoring and state transitions.
UAV_Position	Complex Type (EDouble)	Encapsulates spatial location information, including latitude, longitude, and altitude. Provides high-precision positioning required for sub-meter accuracy navigation.
UAV_Velocity	Complex Type (EDouble)	Represents motion vectors along three axes: VelocityX, VelocityY, and VelocityZ, enabling precise movement tracking and trajectory estimation.
UAV_Orientation	Complex Type (EDouble)	Defines UAV attitude using OrientationPitch, OrientationYaw, and OrientationRoll, which are essential for maintaining stability during coordinated maneuvers.
UAV_Battery	Mixed Type (EBigDecimal, EInt)	Monitors power status using BatteryChargeValue and BatteryChargePercent, enabling fine-grained energy management and operational safety.

**UAV Command and Control Data:** The Command and Control data are important for active mission coordination and real-time management of heterogeneous drones. This enables bidirectional communication between the NDIEM message and the UAVs. The details of each sub-element are presented in Table A3.

**Table A3:** Specification for the UAV_CommandAndControlData.

Attribute	Data Type	Description
commandType	EString/Enum	Specifies the type of command issued to the UAV, including waypoint navigation, flight mode change, emergency handling, and mission update directives.
commandStatus	EString/Enum	Represents the execution state of a command, such as pending, in-progress, completed, or failed, enabling real-time mission tracking.
commandID	EString	A unique identifier for each command instance, ensuring traceability and correct mapping between issued and executed operations.
issueTime	EDate (Optional)	Records the timestamp when the command was generated and dispatched, supporting temporal ordering and synchronization of control operations.
Target_Location	Complex Type (EDouble)	Strongly composed object containing latitude, longitude, and altitude values for precise 3D navigation targeting. This object ensures high-precision waypoint execution and spatial accuracy.

**UAV Sensor Data:** The sensor data represent environmental observations, providing a consistent way to describe raw sensor outputs along with the associated metadata needed for analysis and interoperability, as shown in Table A4.

**Table A4:** Specification for the UAV_SensorData.

Attribute	Data Type	Description
sensorType	EString	Identifies the type of sensor used, such as camera, thermal imager, LiDAR, or magnetometer, enabling multi-modal sensor classification.
sensorID	EString (Optional)	A unique identifier for the sensor unit, supporting traceability in multi-sensor UAV platforms.
UAV_Observation	Complex Type	Represents sensor-generated observations linked to a single sensor source. Each observation is structurally bound to the parent sensor entity.
observationID	EString	A unique identifier for each sensor observation event, ensuring traceability of recorded data instances.
timeStamp	EDate/EInt	Records the exact time of observation, enabling synchronization with UAV telemetry streams.
MediaURL	EString	Stores the reference link to raw sensor data, such as images, video, LiDAR scans, or thermal outputs.
locationOf-Observation	EDouble (Lat, Long, Alt)	Provides the geographical location where the sensor data was captured, enabling spatial contextualization of observations.

**UAV Mission Data:** The mission data defines the overall <missionType> and the specific <missionArea> for the operation. It further comprises mission <objectives>, waypoint sequences, success criteria, and the mission timeline within the collaborative environment, and it is presented in Table A5.

**Table A5:** Specification for the UAV_MissionData

Attribute	Data Type	Description
missionType	EString	Defines the type of mission being executed, such as surveillance, search-and-rescue, mapping, or inspection operations.
missionArea	EString/Geofence Object	Specifies the operational region for the mission, defining spatial boundaries for UAV activity.
Objectives	EString	Represents the high-level goals of the mission, including task definitions and expected outcomes.
WaypointSequence	Complex Type	Defines the ordered sequence of navigation points that the UAV must follow during mission execution.
SuccessCriteria	EString	Specifies the conditions required for successful mission completion, used for evaluation and validation.
missionTimeline	EString/EDate	Defines the temporal structure of the mission, including start time, duration, and scheduling constraints.
Mission-Constraints	Complex Type (EDouble)	Encapsulates operational safety limits, including minAltitude, maxAltitude, and minSpeed, ensuring safe flight execution.

## References

[1] Zhang Z., Wei C., Sheng Y., Zhou Q., Xu J. UAV Interoperability Indicator System and Rating Assessment Methodology. Proceedings of the 2024 Academic Conference of China Instrument and Control Society (ACCIS).

[2] Bitcraze A.B. Crazy Real-Time Protocol (CRTP) Documentation for Crazyflie UAVs. https://www.bitcraze.io/documentation/repository/crazyflie-firmware/master/functional-areas/crtp/.

[3] Ryze Tech Tello EDU SDK 2.0. https://dl-cdn.ryzerobotics.com/downloads/Tello/Tello%20SDK%202.0%20User%20Guide.pdf.

[4] Younas B., Delgado Ruiz J., Ahn S. (2025). Designing an Interoperable UAV Communication Model: A NIEM-Driven Approach. J. Vis. Lang. Comput..

[5] Delgado J., Younas B., Kim J., Ahn S. (2026). Designing a Drone Control Station for Team Missions with Educational Drones. Sensors.

[6] Irfan S., Zhao L., Ullah S., Javaid U., Iqbal J. (2024). Differentiator- and Observer-Based Feedback Linearized Advanced Nonlinear Control Strategies for an Unmanned Aerial Vehicle System. Drones.

[7] Ahn R., Goncalves Junior R., Hill T., Chung L., Supakkul S., Zhao L. Discovering Business Problems Using Problem Hypotheses: A Goal-Oriented and Machine Learning-Based Approach. Proceedings of the 2021 IEEE International Conference on Big Data and Smart Computing (BigComp).

[8] Choi J., Sim T.-S., Hur J.-W. (2025). Development Strategies for Representative Platforms of Defense Unmanned Systems Based on K-MOSA. J. Korea Acad.-Ind. Coop. Soc..

[9] Shakhatreh H., Sawalmeh A.H., Al-Fuqaha A., Dou Z., Almaita E., Khalil I., Othman N.S., Khreishah A., Guizani M. (2019). Unmanned Aerial Vehicles (UAVs): A Survey on Civil Applications and Key Research Challenges. IEEE Access.

[10] Macenski S., Foote T., Gerkey B., Lalancette C., Woodall W. (2022). Robot Operating System 2: Design, architecture, and uses in the wild. Sci. Robot..

[11] Object Management Group (OMG) (2015). Data Distribution Service (DDS) Version 1.4.

[12] MAVLink Development Team MAVLink Micro Air Vehicle Communication Protocol. https://mavlink.io/en/.

[13] PX4 Development Team PX4 Autopilot User Guide. https://docs.px4.io/.

[14] DroneCAN Development Team DroneCAN Specification and Protocol Overview. https://dronecan.github.io/.

[15] NIEM Program Management Office (2024). National Information Exchange Model (NIEM).

[16] (2017). Standard Interfaces of UAV Control System (UCS) for NATO UAV Interoperability.

[17] Neumayr B., Schuetz C.G., Gringinger E., Fabianek C., Vennesland A., Schrefl M., Wilson S. (2021). Providing Packages of Relevant ATM Information: An Ontology-Based Approach. J. Air Transp. Manag..

[18] Axelsson J. (2020). Achieving System-of-Systems Interoperability Levels Using Linked Data and Ontologies. Incose Int. Symp..

[19] Wieczorek J., Bloom D., Guralnick R., Blum S., Döring M., Giovanni R., Robertson T., Vieglais D. (2012). Darwin Core: An evolving community-developed biodiversity data standard. PLoS ONE.

[20] Chapman A.D., Belbin L., Zermoglio P.F., Wieczorek J., Morris P.J., Nicholls M., Schigel D. (2020). Developing standards for improved data quality and for selecting fit for use biodiversity data. Biodivers. Inf. Sci. Stand..

[21] Rosic M., Sumic D., Males L. (2025). Semantic Interoperability of Multi-Agent Systems in Autonomous Maritime Domains. Electronics.

[22] Lan Z., He R., Yu H., Ren Y. Autonomous Transport System Interoperability: Concepts, Technology and Research Prospects. Proceedings of the China Conference on Transportation and Energy Integration Technologies.

[23] FIXM Community (2025). Flight Information Exchange Model (FIXM); Version 4.3.0; FIXM Change Control Board. https://www.fixm.aero.

[24] Lien S.-Y., Deng D.-J., Lin C.-C., Tsai H.-L., Chen T., Guo C., Cheng S.-M. (2020). 3GPP NR Sidelink Transmissions toward 5G V2X. IEEE Access.

[25] Park G., Kim K., Kim J., Jeon H. (2022). The Development Methodology of Korea Command and Control Information Exchange Model. J. Korea Inst. Inf. Commun. Eng..

[26] Shan L., Miura R., Kagawa T., Ono F., Li H.-B., Kojima F. (2019). Machine Learning-Based Field Data Analysis and Modelling for UAV Communications. IEEE Access.

[27] Bray T. (2017). The JavaScript Object Notation (JSON) Data Interchange Format.

[28] Bormann C., Hoffman P. (2020). Concise Binary Object Representation (CBOR).

[29] Google Protocol Buffers Documentation. https://protobuf.dev/.

[30] Fakhreddine C., Raffelsberger M., Sende M., Bettstetter C. Experiments on Drone-to-Drone Communication with Wi-Fi, LTE-A, and 5G. Proceedings of the IEEE Globecom Workshops (GC Wkshps).

[31] Metcalfe M., Nager J., Hacker C.S. (2023). Trust Framework for Data Sharing between Industry and Government. Proceedings of the Integrated Communication, Navigation and Surveillance Conference (ICNS).

[32] Young S., Ancel E., Moore A., Dill E., Quach C., Foster J., Darafsheh K., Smalling K., Vazquez S., Evans E. (2020). Architecture and Information Requirements to Assess and Predict Flight Safety Risks During Highly Autonomous Urban Flight Operations. NASA Technical Reports. https://ntrs.nasa.gov/api/citations/20200001140/downloads/20200001140.pdf.

[33] An D., Joo H., Kim H. (2025). Enabling Low-Latency Digital Twins for Large-Scale UAV Networks Using MQTT-Based Communication Framework. ICT Express.

[34] Hashim H.A. (2025). Advances in UAV Avionics Systems Architecture, Classification and Integration: A Comprehensive Review and Future Perspectives. Results Eng..

[35] Lee W. (2021). Enabling Reliable UAV Control by Utilizing Multiple Protocols and Paths for Transmitting Duplicated Control Packets. Sensors.

[36] Chen H.-H. (2024). Developing a Custom Communication Protocol for UAVs: Ground Control Station and Architecture Design. Internet Things.

[37] Mykytyn P., Brzozowski M., Dyka Z., Langendoerfer P. (2024). A Survey on Sensor- and Communication-Based Issues of Autonomous UAVs. Comput. Model. Eng. Sci..

[38] Xu Y., Hui Z., Deng Y. (2023). Task-Oriented Semantics-Aware Communication for Wireless UAV Control and Command Transmission. IEEE Commun. Lett..

[39] Javaid S., Saeed N., Qadir Z., Fahim H., He B., Song H., Bilal M. (2023). Communication and Control in Collaborative UAVs: Recent Advances and Future Trends. IEEE Trans. Intell. Transp. Syst..

[40] Sayyed A., de Araújo G.M., Bodanese J.P., Becker L.B. (2015). Dual-Stack Single-Radio Communication Architecture for UAV Acting as a Mobile Node to Collect Data in WSNs. Sensors.

[41] Zeng Y., Zhang R., Lim T.J. (2016). Wireless Communications with Unmanned Aerial Vehicles: Opportunities and Challenges. IEEE Commun. Mag..

[42] Younas B. NDIEM: NDIEM-Based Drone Information Exchange Model. https://github.com/B-ushra/NDIEM-A-Networked-Drone-Information-Exchange-Model-for-Heterogenous-Drones.

[43] Ribeiro L.M.B., Müller I., Becker L.B. (2021). Communication Interface Manager for Improving Performance of Heterogeneous UAV Networks. Sensors.

[44] Zager M., Sieber C., Fay A. (2024). Towards Semantic Interoperability: An Information Model for Autonomous Mobile Robots. J. Intell. Robot. Syst..

[45] Nam G., Go J., Kwon C., Jeong S. (2020). A Study on the Analysis and Improvement of STANAG 4586/MAVLink Protocol for Interoperability Improvement of UAS. J. Korea Inst. Mil. Sci. Technol..

[46] Ribeiro L.M.B., Müller I., Becker L.B. (2022). Performance Evaluation of Different ToS Using Heterogeneous Communication Interfaces in FANETs. Front. Future Transp..

[47] Lee K.-H., Oh M., Kim J. (2024). Study on Practical Design of Datalink in Interoperable UAV Systems. J. Korea Inst. Mil. Sci. Technol..

[48] Reichstein L., Schopferer S., Jünger F. Semantic Interoperability of UAV Control Systems Using Ontology and Multi-Protocol Mapping. Proceedings of the International Conference on Unmanned Aircraft Systems (ICUAS).

[49] Zhang S., Zhang H., Song L. (2020). Beyond D2D: Full Dimension UAV-to-Everything Communications in 6G. IEEE Trans. Veh. Technol..

[50] Allouch A., Cheikhrouhou O., Koubâa A., Khalgui M., Abbes T. MAVSec: Securing the MAVLink Protocol for Ardupilot/PX4 Unmanned Aerial Systems. Proceedings of the IEEE The 15th International Wireless Communications & Mobile Computing Conference.

[51] Roy S.K., Samshad M., Rajawat K. (2026). UNet: A Generic and Reliable Multi-UAV Communication and Networking Architecture for Heterogeneous Applications. IEEE Trans. Netw. Serv. Manag..

